# Workload and diagnostic yield of acute neuroradiology scans during on-call hours: past 15-year trends at a European tertiary care center

**DOI:** 10.1007/s00330-025-11881-x

**Published:** 2025-07-30

**Authors:** Kathrin Lamberts, Ömer Kasalak, Maria J. Lamers, Thomas C. Kwee

**Affiliations:** Medical Imaging Center, Department of Radiology, University Medical Center Groningen, University of Groningen, Groningen, The Netherlands

**Keywords:** Computed tomography, Incidental findings, Magnetic resonance imaging, Negative results, Neuroimaging

## Abstract

**Objectives:**

To determine past 15-year trends in workload, negative findings, and incidental findings in acute neuroradiology during on-call hours at a European tertiary care center.

**Materials and methods:**

This study analyzed a sample of 2494 CT and 264 MRI scans of the head and/or neck performed during on-call hours at a tertiary care center on random dates between 2009 and 2023.

**Results:**

The workload significantly increased by 130% between 2009 and 2023 (Kendall tau of 0.76, *p* < 0.001), both due to an increased number and complexity of CT scans (80% and 50% increase, respectively). The workload for MRI remained stable (Kendall tau of 0.03, *p* = 0.869). The proportion of negative CT scans showed temporal stability (Kendall tau of 0.25, *p* = 0.139), at an average of 65%. The proportion of negative MRI scans showed an irregular but significant upward pattern over time, ending at 74% in 2023 (Kendall tau 0.42, *p* = 0.003). The percentage of CT scans with incidental findings exhibited a fluctuating yet significant upward trend over time, ending at 4% in 2023 (Kendall tau 0.62, *p* < 0.001), and was associated with increasing age (*p* = 0.010) and the use of a larger scan coverage (*p* < 0.001). No incidental findings were reported for MRI.

**Conclusion:**

The acute neuroradiology workload during on-call hours has considerably grown in the past 15 years, both due to a rise in the number and complexity of CT scans. Overuse of CT did not appear to increase, but MRI may be increasingly overutilized. The frequency of incidental findings on CT was non-negligible and increased.

**Key Points:**

***Question*** *Both an increase in the number and complexity of CT scans has led to a 130% rise in acute neuroradiology workload during on-call hours over the past 15 years*.

***Findings*** *There are indications that MRI overuse may be increasing (but not CT), while the frequency of incidental findings on CT is also on the rise*.

***Clinical relevance*** *Policymakers should address the rising acute neuroradiology workload driven by CT and the potential overuse of MRI in this setting. Further investigation is needed into the consequences of the increase in incidental findings on CT*.

**Graphical Abstract:**

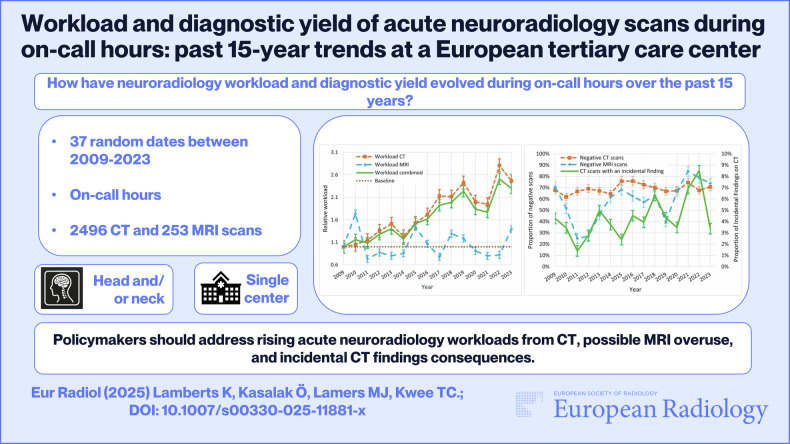

## Introduction

The use of medical imaging has increased considerably over the past decades, and is expected to keep on growing in the foreseeable future [1–3]. Neuroimaging utilization has faced a similar surge [2, 4, 5].

The reported imbalance between the demand for imaging and the available radiology workforce has sparked discussions on the risk of radiologist burnout and the quality of radiology service that can be delivered [6–8]. During on-call hours, the pressure on radiology departments may be particularly high, because there is usually less staff available to process the clinical demand. Currently, there is a lack of clear data on how much the neuroradiological workload during on-call hours has increased over the past years. This information is important for departmental managers and hospital administrations to understand if, and how much, additional staff should be hired.

Overuse, defined as a health care service that is provided under circumstances in which its potential for harm exceeds the possible benefit [9], is a potential cause of the increased use of medical imaging. Imaging overuse is driven by several factors, including defensive medicine/fear of malpractice, the presence of less experienced staff, easy access to imaging, pressure from patients, a lack of time to examine patients, pressure from consultants to perform imaging, the use of imaging to decrease turnaround time in the emergency department, and a lack of proper medical education [10]. If overuse were indeed to contribute to the rise in medical imaging utilization, we would expect the proportion of negative examinations to increase over time. However, it is currently unclear if this is the case for acute neuroradiology.

Another important issue to consider is whether the number of incidental imaging findings (defined as an imaging abnormality in a symptomatic patient, where the abnormality was not apparently related to the patient’s symptoms [11]) has increased because of the increased use of acute neuroimaging. Incidental findings generally result in low-value and potentially harmful care with associated costs [12]. If the number of incidental findings is found to be increasing, this should alert physicians and policymakers to apply more stringent criteria for acute neuroimaging.

The purpose of this study was therefore to determine past 15-trends in workload, negative findings, and incidental findings in acute neuroradiology during on-call hours at a European tertiary care center.

## Materials and methods

### Study design

This retrospective, cross-sectional study was conducted at the University Medical Center Groningen (a tertiary care center in the Netherlands), with local institutional review board ethical approval and a waiver of informed consent. This study assessed the use of urgent CT and MRI scans of the head and neck during evening and night duty shifts from 2009 to 2023. It analyzed temporal trends in workload, negative scans, incidental findings, and associated determinants.

### Data selection

A sample of 37 unique calendar dates (10% of all days per year) was randomly chosen. All urgent head and/or neck CT and MRI scans performed during on-call hours from 17.00 pm to 8.00 am, performed on all of these 37 calendar days (including weekend days) in each year between 2009 and 2023, were included. Scans performed for elective purposes, scans only performed for treatment planning and not for diagnostic purposes (e.g., preoperative CT or MRI for neuronavigation), scans without any clinical information or a radiology report, scans performed for research purposes, and uninterpretable scans (e.g., due to severe motion artefacts) were excluded. Note that at our hospital, all head and/or neck CT and MRI scans are either interpreted by neuroradiologists or reviewed under their supervision when residents perform the initial reading.

### Data extraction

For each scan that was included the following data were collected: patient’s age and gender, indication for the scan (categorized as stroke, trauma, acute oncology, inflammation/infection, hydrocephalus, and other), scan protocol, anatomical region (head only, neck only, or head and neck), workload expressed in relative values units (Relative value units (RVUs), according to national guidelines [13] [Supplementary Table 1]), scan result, and whether an incidental finding was present. CT scan protocols were classified into three categories: (1) ”head only” (either unenhanced or contrast-enhanced), (2) “stroke combination” (unenhanced CT of the head, CT angiography [CTA], and CT perfusion), and (3) “head and neck” (either unenhanced or contrast-enhanced). MRI scan protocols were not classified due to the relatively low sample of MRI scans. The scan result was classified as either positive (i.e., new findings which are a reasonable explanation for the patient’s complaints or symptoms), negative (i.e., the absence of findings related to the reason that the scan was made and no disease deterioration or other new and clinically relevant findings compared to a previous imaging examination when available) [14], or indeterminate. All incidental findings (i.e., an imaging abnormality of clinical relevance or unknown relevance in a symptomatic patient, where the abnormality is not apparently related to the patient’s symptoms [11]) were counted, with the exception of findings such as degenerative changes, sinusitis, and thyroid nodules, as these conditions are common and less likely to significantly impact clinical management or represent newly discovered pathology in the emergency setting [15–17].

### Statistical analysis

The average number of scans and workload, proportion of negative scans (indeterminate scan results excluded), and incidental findings per duty shift per year were calculated, and temporal trends between 2009 and 2023 were assessed using Mann–Kendall tests and Kendall’s correlation. Logistic regression analysis was performed to assess the association between a negative scan result and with patient’s age, gender, and indication for the scan. Logistic regression was also used to determine if the presence of incidental findings was associated with patients’ age and CT scan protocol. *p*-values < 0.05 were considered statistically significant. Statistical analyses were conducted using Microsoft Excel (version 2408) with the Real Statistics Resource Pack (version 9.2.2) and IBM SPSS (version 28).

## Results

### Scans and patient characteristics

**Fig. 1:**
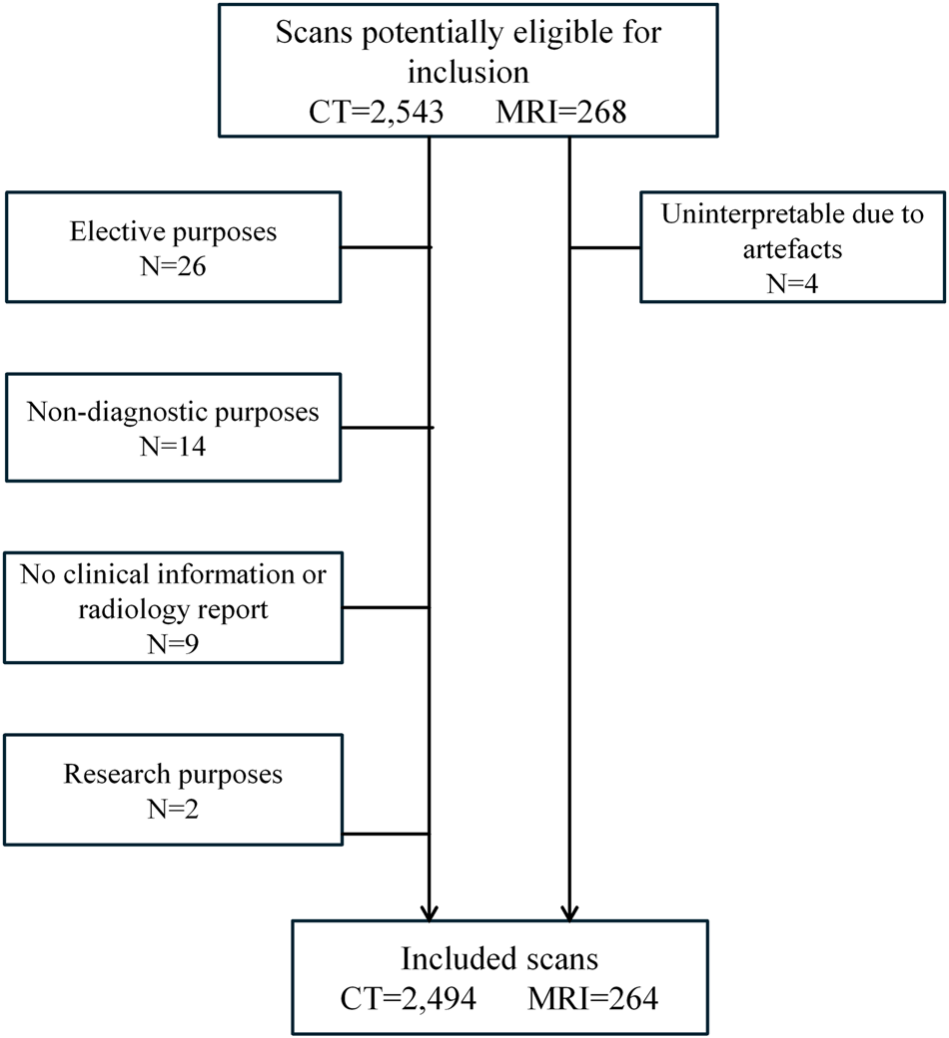
Inclusion flowchart

A total of 2543 CT scans and 268 MRI scans were performed during on-call hours on the 37 randomly sampled calendar days between 2009 and 2023, of which 2494 CT scans and 264 MRI scans were included (Fig. 1). Patient characteristics, scan indications, and anatomical regions are summarized in Table 1. The average patient age increased significantly during the study period from 47.3 to 48.5 years (Kendall tau 0.41, *p* = 0.041). The most common indication for CT was stroke (43%), followed by trauma (40%), other/mixed (8%), hydrocephalus (4%), acute oncology (3%), and infection/inflammation (2%). For MRI, stroke was also the leading indication (42%), followed by other/mixed (24%), acute oncology (12%), hydrocephalus (11%), infection/inflammation (8%), and trauma (2%). The distributions of indications for both CT and MRI did not significantly change over time (*p* = 0.255 and *p* = 0.079, according to the Mann–Kendall test and Fisher’s method, respectively).

**Table 1:** Patient characteristics, scan indications, and anatomical regions

Category	CT (*n* = 2494)	MRI (*n* = 264)
Patient characteristics		
Age (years)	54.9 ± 22.4	42.8 ± 22.9
Age range	1 month to 98 years	4 days to 89 years
Age < 18 years	124	46
Age < 28 days	1	4
Male/female	1432/1062	141/123
Scan indications		
Stroke	43%	42%
Trauma	40%	2%
Acute oncology	3%	12%
Inflammatory/infection	2%	8%
Hydrocephalus	4%	11%
Other	8%	24%
Anatomical regions		
Head only	63%	93%
Neck only	1%	0%
Head and neck	36%	7%

### Workload

**Fig. 2:**
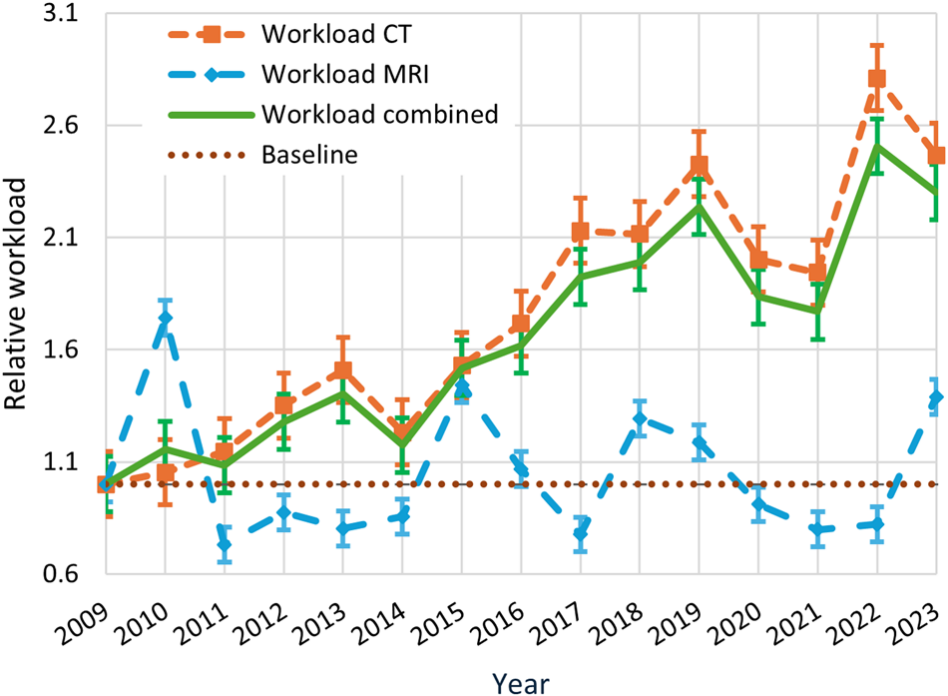
Relative workload for urgent CT and MRI scans during on-call hours on 37 randomly sampled distinct calendar days in each year between 2009 and 2023, calculated using RVUs. The workload for each year was standardized in this figure, using the baseline year of 2009 as the reference point. A value below 1 indicates a lower workload compared to 2009, while a value above 1 reflects a higher workload. For instance, a value of 2.0 in a given year indicates that the workload during on-call hours was twice as high as in 2009. The standard deviations for each data point are also provided

**Fig. 3:**
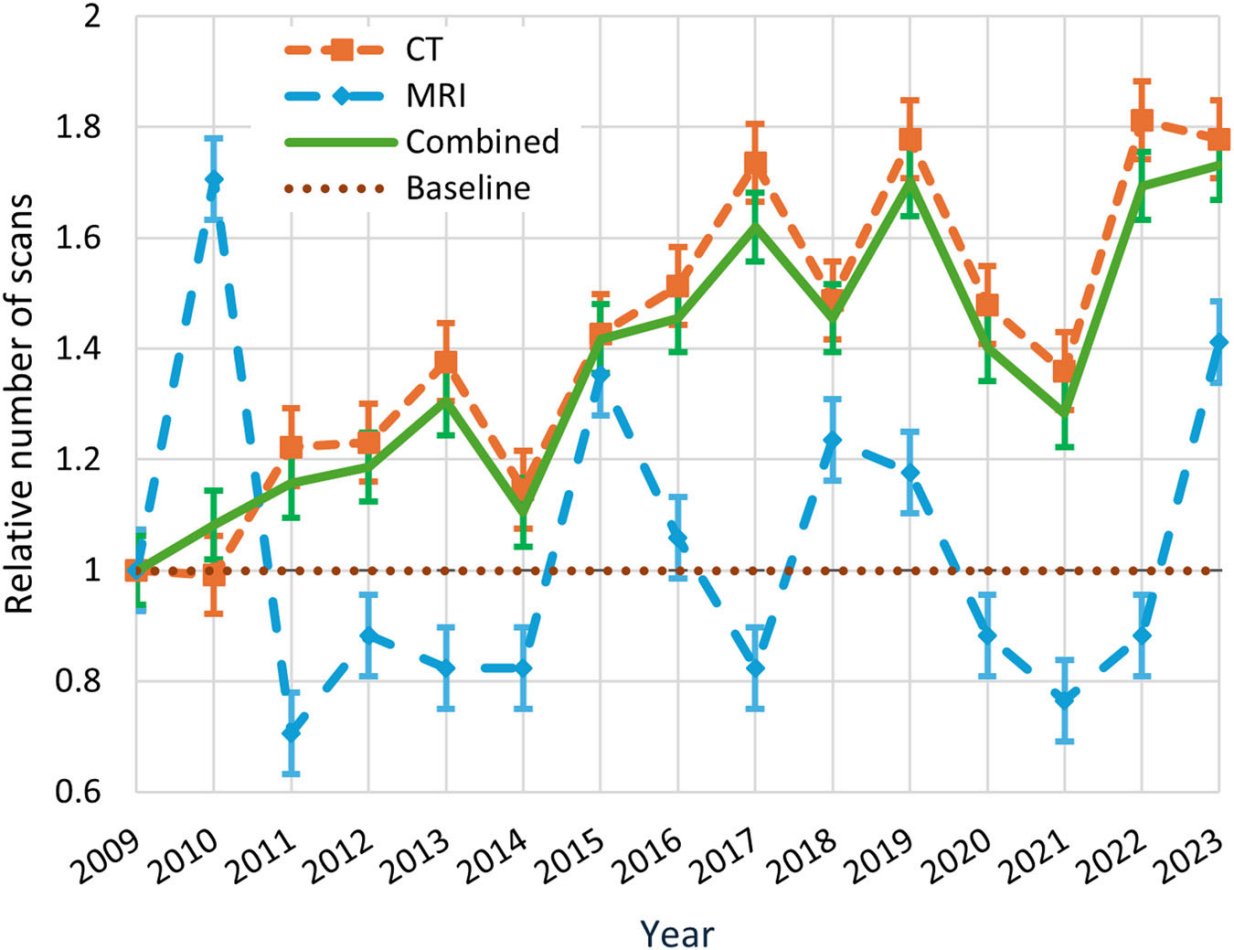
Relative number of urgent CT and MRI scans during on-call hours on 37 randomly sampled distinct calendar days in each year between 2009 and 2023. The data for each year was normalized using 2009 as a reference. Values below 1 indicate a reduction in the number of examinations relative to 2009, while values above 1 reflect an increase. For instance, a value of 2.0 in a given year indicates that the number of scans during on-call hours was twice as high as in 2009. The standard deviations for each data point are also provided

**Fig. 4:**
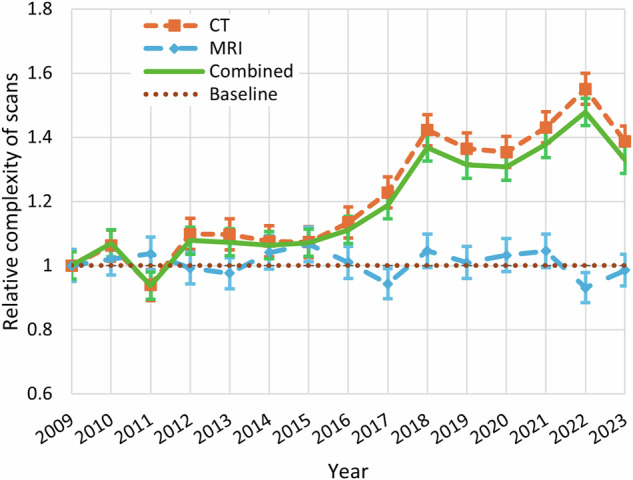
Relative complexity per urgent CT and MRI scan during on-call hours on 37 randomly sampled distinct calendar days in each year between 2009 and 2023, calculated as the total number of RVUs divided by the total number of examinations in each year. The data for each year was normalized using 2009 as a reference. Values below 1 indicate a reduction in the complexity of examinations relative to 2009, while values above 1 reflect an increase. For instance, a value of 2.0 in a given year indicates that the complexity of scans during on-call hours was twice as high as in 2009. The standard deviations for each data point are also provided

The relative workload significantly increased by 130% between 2009 and 2023 (Kendall tau of 0.76, *p* < 0.001) (Fig. 2). For CT, the workload increased by 130% (Kendall tau of 0.76, *p* < 0.001), while the workload for MRI did not significantly change between 2009 and 2023 (Kendall tau of 0.03, *p* = 0.869). The increased workload related to CT usage was attributable to both an increased number (80% increase, Kendall tau of 0.63, *p* < 0.001) and complexity (50% increase, Kendall tau of 0.71, *p* < 0.001) of CT scans (Figs. 3 and 4). Notably, the proportion of CT scans of the head only decreased significantly from 79% in 2009 to 50% in 2023 (Kendall tau of −0.69, *p* < 0.001). Meanwhile, the CT stroke combination protocol usage increased from 6% in 2009 to 18% in 2023 (Kendall tau of 0.76, *p* < 0.001). The use of head and neck CT scans also increased from 15% to 30% during this same time period, although not significantly (Kendall tau of 0.34, *p* = 0.089).

### Negative scan proportion

**Fig. 5:**
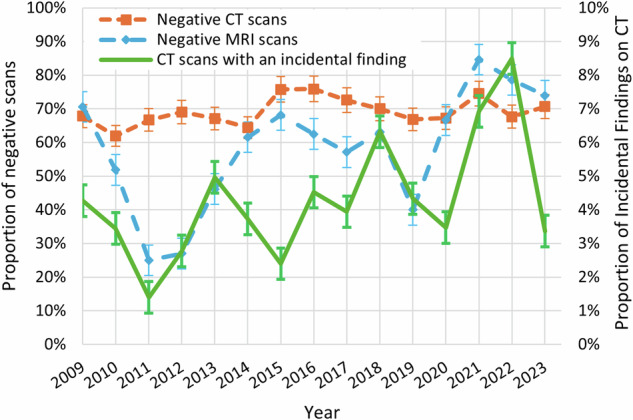
Proportions of negative CT and MRI scans and proportion of CT scans with an incidental finding during on-call hours on 37 randomly sampled distinct calendar days in each year between 2009 and 2023. The standard deviations for each data point are also provided

The proportion of negative CT scans remained stable over the years (Kendall tau of 0.25, *p* = 0.139), at an average of 65% (Fig. 5). The proportion of negative MRI scans (the main indications were stroke (26%) and other/mixed (16%)) showed an irregular but significant upward pattern over time, ending at 74% in 2023 (Kendall tau 0.42, *p* = 0.003) (Fig. 5). There was no significant association between negative CT or MRI scans and patient age, gender, or indication for the scan on logistic regression analysis (*p* = 0.318, *p* = 0.090, *p* = 0.364, respectively).

### Incidental findings

The percentage of CT scans with incidental findings exhibited a fluctuating yet significant upward trend over time, ending at 4% in 2023 (Kendall tau 0.62, *p* < 0.001) (Fig. 5). The most common locations for incidental findings were intracranial (44%) and lungs (27%) (Table 2). For intracranial findings, the most common incidental findings were meningioma (10 cases), arachnoid cyst (8 cases), and colloid cyst (3 cases) (Table 2). In the lungs, incidental findings included nodules (10 cases) and pleural effusions (6 cases) (Table 2). The presence of incidental findings on CT was significantly associated with increasing age (odds ratio (OR) of 1.01 [95% confidence interval (CI): 1.00–1.02] per additional year of age, *p* = 0.010) and the use of a larger scan coverage (ORs of 2.94 [95% CI: 1.80–4.81] for “stroke combination” and 4.87 [95% CI: 3.19–7.44] for “head and neck” protocols, compared to “head only” protocols, *p* < 0.001) on multivariate analysis. No incidental findings were reported for MRI (note that 76% of patients who underwent MRI had previously undergone either CT or MRI).

**Table 2:** Overview of 106 incidental findings on 2494 urgent CT scans of the head and/or neck on 37 randomly sampled distinct calendar days for all years between 2009 and 2023

Incidental finding	No.	%
Periodontal	12	11%
Pulmonary nodule	10	9%
Meningioma	10	9%
Arachnoid cyst	8	8%
Intracranial aneurysm	8	8%
Pleural effusion	6	6%
Pneumothorax	5	5%
Skull lesions	5	5%
Cerebral metastasis	4	4%
Colloid cyst	3	3%
Other intracranial abnormalities^1^	13	12%
Other pulmonary abnormalities	6	7%
Other vascular abnormalities	5	5%
Other abnormalities^2^	11	10%

^1^ a.o. mega cisterna magna (*n* = 2), cystic abnormalities unspecified (*n* = 4)

^2^ a.o. parotid lesions (*n* = 2), enlarged thyroid (*n* = 1), lipoma (*n* = 1), mediastinal struma (*n* = 1)

## Discussion

The results of our study show that the acute neuroradiology workload during on-call hours has considerably increased (130%) over the past 15 years. This rise in workload is attributable to both an increase in the volume of patients requiring urgent CT, and a shift towards more complex CT protocols, particularly with the growing use of stroke combination protocols (comprising unenhanced CT of the head, CTA, and CT perfusion). Therefore, neuroradiologists not only have to manage more cases but also more challenging and time-consuming studies during on-call hours. Policymakers must ensure that the increased workload is adequately compensated for by a proportional increase in the number of neuroradiologists, in order to keep the healthcare demand manageable. Notably, despite the marked increase in both volume and complexity of imaging during on-call hours, the staffing per shift has remained stable over the past 15 years. It remains unclear how increasing staffing would affect individual workload, as reduced pressure per radiologist might be counterbalanced by a higher volume of scan requests due to increased availability. To date, there is a lack of scientific literature examining individual workload intensity during on-call hours, particularly studies that incorporate both image interpretation and non-interpretative tasks such as answering telephone calls, consulting with referring physicians, performing administrative duties, and arranging imaging logistics. Future research is needed to establish benchmarks for workload in emergency neuroradiology, which could guide staffing decisions and improve working conditions. Importantly, our results also emphasize that RVUs, rather than the number of exams performed, should be used to monitor workloads and plan staff capacity, as the latter may underestimate the actual workload.

The rise in the number and complexity of scans may be caused by increased reliance of referring physicians on neuroimaging, the recommendation to use (advanced) imaging techniques in guidelines that have been published over the past years (such as for acute stroke and trauma [18, 19]), the aging population, and our institution’s growing emphasis on high-complexity care in recent years (which often demands more advanced diagnostic examinations). Interestingly, the proportion of negative CT scans remained relatively stable over the past 15 years, which argues against overutilization of CT. However, the percentage of negative MRI scans has significantly increased over the years, suggesting that MRI is being used more frequently to exclude disease—an indication of potential overuse. However, it should be noted that negative scans (such as the 26% of negative MRI scans that were performed for stroke evaluation in our analysis), can still be valuable to rule out acute pathology and aid in clinical decision-making.

Importantly, the increased use of CT scans during on-call hours led to more incidental findings, particularly due to the aging population and the use of larger scan coverage, which seems to be a plausible explanation. Whether these incidental findings were clinically relevant was outside the scope of this study, but their frequency (5%) is sufficiently high to warrant consideration by referring physicians when ordering scans. Interestingly, no incidental findings were reported for any of the MRI scans that were included in this study. This can be explained by the fact that most of these patients (76%) had previously undergone either CT or MRI, and the new MRI scan did not detect any new incidental findings that could be counted.

Several previous studies investigated workload trends in the neuroradiology setting. Verdoorn et al [20] reviewed all overnight neuroradiology CT scans that were primarily read by residents in a single center in the United States, and reported that the mean number of cases per night during the weekday increased from 3.0 in 2006 to 5.2 in 2010 [20]. During the weekend, the mean number of cases per night increased from 5.4 in 2006 to 7.6 in 2010 (*p* < 0.001) [20]. Allen et al [4] analyzed radiology trainee neuroimaging workloads nationwide, using Medicare claims files from 2002 to 2018. Mean annual per radiology trainee neuroimaging Medicare RVUs increased 174.9% between 2002 and 2018 (4). Mean per trainee RVU increases were greatest for spine CT (394.2%) but present across all neuroimaging services [4]. In another study, Bruls et al [1] analyzed the workload for radiologists during on-call hours in a large general hospital in the Netherlands between 2006 and 2020. They reported that the overall workload in terms of RVUs during on-call hours had quadrupled, primarily due to the increased use of CT. CT examinations of the head, neck, and spine were among the CT studies that increased by more than 500% between 2006 and 2020 [1]. The increase in neuroradiology exam volumes that was reported in the studies by Verdoorn et al [20], Allen et al [4], and Bruls et al [1] is in line with our results. However, these previous studies did not provide data on how much the neuroradiological workload during on-call hours had actually increased over the past years in terms of RVUs. Our study showed that a significant increase in more complex neuroradiology examinations was primarily responsible for the 130% increase in workload in terms of RVUs between 2009 and 2023. Furthermore, these previous studies analyzed trends in negative scan proportions and incidental findings.

The present study had some limitations. First, our study was performed at a tertiary care academic hospital in Western Europe. Our findings may not necessarily be the same in non-academic hospitals and in other regions, as the proportion of negative scans, availability of subspecialized neuroradiologists, and imaging decision-making dynamics might differ in smaller or non-academic hospitals. Of note, our data show that the increasing neuroradiology workload during shift hours is primarily driven by CT, while MRI utilization has remained relatively stable. This likely reflects local clinical protocols and logistical limitations, as MRI is less frequently used for emergency indications at our center. However, recent studies from other countries indicate a growing use of emergency MRI, particularly in acute stroke care [21–23], highlighting that imaging trends may vary across institutions and healthcare systems. Further studies are therefore needed to assess the generalizability of our findings. Third, nationally established RVUs were used to calculate workload. However, RVUs do not account for the number of slices and resolution of imaging studies, which have increased over the years with advancing technology and may have further contributed to the workload [24]. Fourth, we were unable to quantify the additional workload in terms of actual time spent during on-call hours. The time required per scan varies widely due to factors beyond image interpretation, including non-interpretative tasks and interruptions such as being woken by urgent calls. These complexities make retrospective estimation of workload in minutes challenging and may underestimate the true impact on radiologists’ rest and performance. Fifth, the proportion of negative scans was interpreted as an indicator of imaging overuse. However, the definition of “overuse” and the acceptable proportion of negative scans remain unclear [10]. Sixth, we did not investigate if the incidental findings in our study actually led to low-value and potentially harmful care with associated costs.

In conclusion, the acute neuroradiology workload during on-call hours has considerably grown in the past 15 years, both due to a rise in the number and complexity of CT scans. Overuse of CT did not appear to increase, but MRI may be increasingly overutilized. The frequency of incidental findings on CT was non-negligible and increased.

## Supplementary information

ELECTRONIC SUPPLEMENTARY MATERIAL

## Abbreviations

CI: Confidence interval
CTA: CT angiography
OR: Odds ratio
RVU: Relative value unit
