# Supplementary material for: Workload and diagnostic yield of acute neuroradiology scans during on-call hours: past 15-year trends at a European tertiary care center
Source: Eur Radiol. 2025 Jul 30;36(1):775–82. doi: 10.1007/s00330-025-11881-x (PMC12711919; doi:10.1007/s00330-025-11881-x)
Supplement: Supplementary file 1 — ELECTRONIC SUPPLEMENTARY MATERIAL [file 330_2025_11881_MOESM1_ESM.pdf]

Workload and diagnostic yield of acute neuroradiology scans during on-call hours: past 15-year trends at a European tertiary care center

ELECTRONIC SUPPLEMENTARY MATERIAL

Supplementary Table 1. RVUs of scan protocols used in this study, according to national guidelines [13].

Type of scan	Description	RVUs
CT	Brain and/or skull with or without contrast agent[1]	12
CT	Spine (partial scan)	15
CT	Neck including CTA neck[1]	30
MRI	Cerebrum – standard	18
MRI	Cerebrum – with contrast agent	21
MRI	Epilepsy	26.25
MRI	Posterior cranial fossa	15

Notes:
[1] For stroke patients, in whom CT cerebrum, CTA carotids, and CT perfusion are performed, CT head can be registered together with CT neck, if the carotids are also imaged. There are no separate RVUs for CT perfusion [13].
